# Unusual Presentation of Vivax Malaria with Anaemia, Thrombocytopenia, Jaundice, Renal Disturbance, and Melena: A Report from Malang, a Nonendemic Area in Indonesia

**DOI:** 10.1155/2013/686348

**Published:** 2013-12-29

**Authors:** Loeki Enggar Fitri, Teguh Wahju Sardjono, Bagus Hermansyah, Didi Candradikusuma, Nicole Berens-Riha

**Affiliations:** ^1^Department of Parasitology, Faculty of Medicine, University of Brawijaya, Jalan Veteran Malang, East Java 65145, Indonesia; ^2^Department of Parasitology, Faculty of Medicine, University of Jember, Jalan Kalimantan 37 Kampus Tegalboto, Jember East Java 68121, Indonesia; ^3^Department of Internal Medicine, Dr. Saiful Anwar Hospital/Faculty of Medicine, University of Brawijaya, Jalan Veteran Malang, East Java 65145, Indonesia; ^4^Division of Infectious Diseases and Tropical Medicine, Medical Center of the University of Munich (LMU), Leopoldstrasse 5, 80802 Munich, Germany

## Abstract

Most of the complications of malaria such as anaemia, thrombocytopenia, jaundice, and renal failure are commonly found in *Plasmodium falciparum* malaria, but the incidence of severe and complicated vivax malaria tends to be increasing. We report two cases of severe *Plasmodium vivax* malaria from Malang, a nonendemic area in Indonesia. Patients exhibited anaemia, thrombocytopenia, jaundice, renal disturbance, and melena. Microscopic peripheral blood examination and amplification of parasite 18s rRNA by polymerase chain reaction showed the presence of *P. vivax* and absence of *P. falciparum*. All patients responded well to antimalarial drugs.

## 1. Introduction 

Several hepatic and renal complications are associated with complicated and severe falciparum malaria. Of the five human pathogenic species, *Plasmodium falciparum* is the most dangerous, since it can cause more severe manifestations and multisystem organ failure. Previously, *Plasmodium vivax* was thought as a benign parasite causing nonsevere and uncomplicated malaria, but recent reports about life-threatening complications from endemic regions such as Ethiopia [1], India [2–5], Brazil [6], and Papua Indonesia [7, 8] implicate that vivax malaria was dangerously underestimated. *Plasmodium vivax* seems to cause complications of severe malaria, including cerebral malaria [2–5], renal failure [5], circulatory collapse [5], pulmonary dysfunctions [5, 9] liver dysfunction [4, 5], thrombocytopenia [4], and severe anaemia [5].

## 2. Case Presentation

### 2.1. Case 1

**Figure 1:**
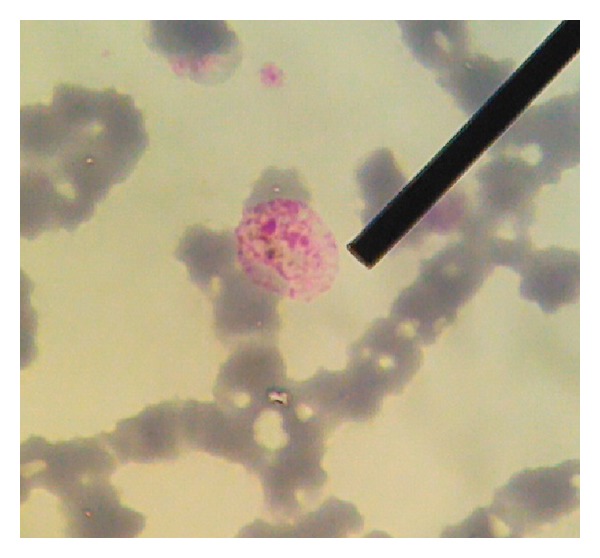
Peripheral blood smear of case 1 showed gametocyte form of *Plasmodium vivax.*

A 28-year-old female presented with high and intermittent fever for more than three days, chills, nausea, cold sweat, and heartburn but no bleeding. Last year, she worked in Papua and was twice hospitalised because of malaria. Physical examination showed a GCS score of 15, blood pressure of 110/80 mmHg, pulse rate of 80/min, respiratory rate of 16/min, and body temperature at arrival of 36.5°C. Laboratory evaluation showed haemoglobin (Hb) of 8 mg/dL, leucocyte count of 4890/mm^3^, platelet count of 42000/mm^3^, glucose of 89 mg/dL, urea of 18.8 mmol/L, and serum creatinine of 0.56 mg/dL. The liver function tests showed an AST of 24 IU/L, ALT of 24 IU/L, total bilirubine of 2.59 mg/dL, direct bilirubine of 1.3 mg/dL, and indirect bilirubine of 1.25 mg/dL. Serum electrolyte analysis showed low potassium (2.94 mmol/L) and normal sodium levels (136 mmol/L). Blood smear showed *Plasmodium vivax *(parasitaemia 0.6%) as well as PCR result (Figure 1). No seizures or cerebral symptoms were observed. Patient received oral quinine 3 × 400 mg/day for 7 days and primaquine therapy 1 × 15 mg/day for 14 days. She decided to leave the hospital before total recovery (Table 1).

### 2.2. Case 2

**Figure 2:**
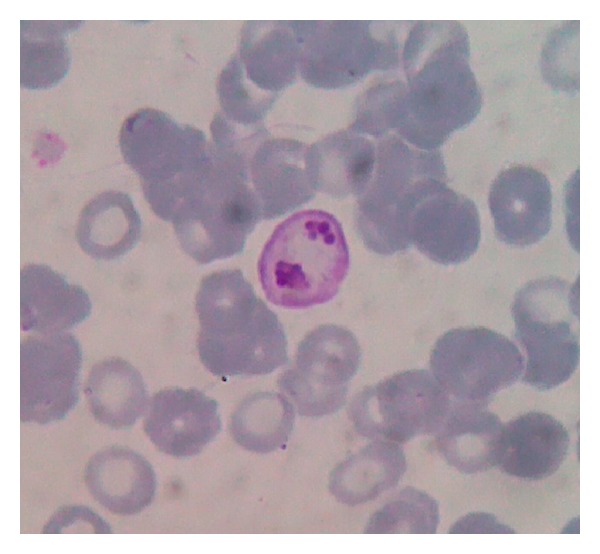
A peripheral blood smear of case 2 showed immature schizonts form of *Plasmodium vivax.*

A 29-year-old pregnant female had fever 9 days before admission, also chills, nausea, vomiting, and melena. On physical examination, she showed a GCS score of 15, blood pressure of 110/80 mmHg, pulse rate of 90/min, respiratory rate of 20/min, and body temperature at arrival of 38.3°C. Laboratory evaluation showed a Hb of 6.9 mg/dL, leucocytes of 5870/mm^3^, platelet count of 54000/mm^3^, glucose of 68 mg/dL, urea of 77.7 mmol/L, serum creatinine of 1.46 mg/dL, AST of 354 IU/L, ALT of 88 IU/L, albumin of 1.88 g/L, total bilirubine of 4.61 mg/dL, direct bilirubine of 3.27 mg/dL, and indirect bilirubine of 1.34 mg/dL. Serum electrolyte analysis showed normal values. No seizures or cerebral symptoms were observed. Based on PCR results, the patient was positive for *Plasmodium vivax* confirming the microscopic diagnosis (parasitaemia 0.3%) (Figure 2). The patient lived in Papua since 1.5 years. She received oral arterakine (dihydroartemisinin 40 mg/piperaquine 320 mg) therapy 4 × 1 tablet/day and recovered after 12 days (Table 1). There were no data recorded for the baby.

## 3. Discussion

Malang is known to be a nonendemic area of malaria in Indonesia, but some of the imported cases of malaria admitted to the Dr. Saiful Anwar Hospital showed severe clinical and laboratory complications. All patients in this study were admitted to the emergency unit. Clinical and laboratory examinations including Giemsa thin smear and polymerase chain reaction (PCR) were targeted against the 18S rRNA gene of the parasite were conducted to confirm the diagnosis [10].

Both cases had moderate anaemia with haemoglobin levels <10 mg/dL (based on WHO criteria, severe anaemia Hb <5 mg/dL). Anaemia is a common and frequently severe consequence of *vivax* infection. Females are at greater risk of hospitalization with *Plasmodium vivax* malaria than males and in one large analysis were more likely to present with anaemia. Pregnant women with *Plasmodium vivax* infection have a 2-fold higher risk of moderate anaemia than uninfected [11, 12]. In malaria, low haemoglobin may result from acute haemolysis due to destruction of both infected and uninfected red blood cells and dyserythropoiesis. In pregnant women, anaemia is also correlated with nutritional deficiencies [12]. The mechanism of malaria associated with severe anaemia is multifactorial and include intensive haemolysis of circulating infected RBCs and noninfected erythrocytes due to glycosylphosphatidyl-inositol toxin release as wells as dyserythropoiesis due to cytokine effects and other inducers of inflammation such as haemozoin [13]. The primary target of human *Plasmodium* species is the red blood cell. *Plasmodium vivax *has a very strong predilection for young red blood cells that have emerged from the bone marrow within the last 14 days. Despite reaching lower densities than *P. falciparum*, *P. vivax* causes similar absolute reduction in red cell mass because it results in proportionately greater removal of uninfected red blood cells [11].

Mild hypoglycaemia occurred in the second case (WHO criterion for severe hypoglycaemia is <40 mg/dL). As the patient was pregnant, hypoglycaemia could also be due to pregnancy [12]. Malaria associated hypoglycaemia due to increased metabolic demands of febrile illness, parasite utilization of glucose, failure of hepatic gluconeogenesis, and glycogenolysis [14]. More likely, the hypoglycaemia condition can be a result of a quinine-induced hyperinsulinaemia for the patient who receive quinine, a drug that is well known for causing hypoglycaemia [15].

The first case had a platelet count less than 50000/mm^3^. Severe thrombocytopenia is common in isolated *falciparum* and mixed *falciparum/vivax *malaria but is very rare in isolated *Plasmodium vivax* infection. The mechanism of thrombocytopenia in malaria is uncertain. In vivax infection, it may be due to direct lysis of the platelets, reduced platelet survival, and IgG mediated destruction [4, 16].

The incidence of leucopenia in this study was consistent with other reports. The leukocyte count in malaria is from low to normal due to the localisation of leukocytes away from peripheral circulation to spleen and other marginal pools rather than actual depletion or stasis. This is a transient finding like thrombocytopenia and normalises after antimalarial therapy [17].

The current study documented the severe manifestations of *Plasmodium vivax* malaria based on WHO criteria (jaundice, acute renal disturbance, and hepatic dysfunctions). Malaria-associated renal and hepatic dysfunctions are severe complications that are increasingly becoming a problem of great concern in malaria endemic countries. According to the World Health Organization, serum creatinine levels above 4.77 mg/dL are an indication of acute renal failure. Serum creatinine level in the second case showed only acute renal disturbance. The mechanisms proposed for acute renal disturbance in *Plasmodium vivax* infection are heavy parasitaemia, acute tubular necrosis, volume depletion, intravascular haemolysis, renal ischemia, and microvascular sequestration [18].

The increase of serum level of hepatic enzymes, transaminases (AST and ALT), and alkaline phosphatase is the markers of liver damage [19]. The second case showed elevated AST and ALT levels. Jaundice in malaria may be explained by severe haemolysis (indirect bilirubine predominance) or liver cholestasis (direct bilirubine predominance). Causes of jaundice in malaria can be classified into direct causes, including malarial hepatitis and intravascular haemolysis of parasitized red blood cells as well as indirect causes including microangiopathic haemolysis associated with disseminated intravascular coagulation (DIC), G6PD-related haemolysis, antimalarial drug induction, septicemic hepatitis, and unrelated causes such as coexisting acute viral hepatitis [19, 20].

Gastrointestinal bleeding in the form of melena occurred in the second case. The bleeding can occur for many reasons, one of which is the DIC. Cases of melena in *Plasmodium vivax* infection have also been reported by Lakhar et al., (1996) who explained that DIC is a major cause of bleeding [21]. Platelet count was low in both cases but above 10,000/*μ*L. Acute bleeding due to thrombocythopenia is unlikely as long as platelet function is still effective.

*Vivax* malaria has been long considered to have a benign course. It is known for multiple relapses, but the typical complications seen with falciparum malaria are not found with vivax monoinfection. However, in the past few years severe and complicated clinical manifestations of vivax malaria are frequently reported, sometimes even fatalities [22, 23]. In this study it was shown that *Plasmodium vivax* malaria can also cause severe complications such as anaemia, thrombocythopenia, melena, acute renal disturbance, hepatic dysfunction, and jaundice. Pregnancy in *Plasmodium vivax* infection is clearly associated with serious complication as mentioned in this case and reported previously [12]. Rapid diagnostic tests result in prompt diagnosis, but microscopic and if possible molecular confirmation is necessary to safely diagnose mixed infections with other species. Intensive care and supportive measures along with standard protocols of management are required to treat these cases. Clinicians must be aware of unusual manifestations and prompt presentation of vivax malaria infection as mentioned in this case report and others. Early diagnosis and treatment can minimize associated morbidity and mortality. Due to changes in epidemiology, recognition of severe manifestations of *Plasmodium vivax* malaria, and emerging drug resistance, emphasis must be on strict preventive measures to lower the burden of this disease [24].

## Figures and Tables

**Table 1:** Description data of two cases.

	Case 1	Case 2
Age	28 yrs	29 yrs
Sex	F	F
Fever	>3 days	9 days
Cerebral symptoms/GCS	Absent	Absent
Haemoglobin (mg/dL)	8*	6.9*
Leucocytes (cell/mm^3^)	4890	5870
Platelets (cell/mm^3^)	42000*	54000*
Blood glucose (mg/dL)	89	68
Urea (mmol/L)	18.8	77.7**
Serum creatinine (mg/dL)	0.56	1.46**
AST (IU/L)	24	354**
ALT (IU/L)	24	88**
Albumin (g/L)	—	1.88
Direct bilirubine (mg/dL)	1.3**	3.27**
Indirect bilirubine (mg/dL)	1.25**	1.34**
Total bilirubine (mg/dL)	2.59**	4.61**
Serum electrolyte analysis	Na: 136, K: 2.94* and Cl: 108	Na: 135, K: 3.88 and Cl: 113
Peripheral smear	*P. vivax *	*P. vivax *
Nested PCR	*P. vivax *	*P. vivax *
Drug given	Oral quinine and primaquine	Oral arterakine (dihydroartemisinin + piperaquine)
Hospitalisation	Discharged before fully recovered	12 days

Description: *low, **high.
